# Prognostic value of CT-based radiomics in grade 1–2 pancreatic neuroendocrine tumors

**DOI:** 10.1186/s40644-024-00673-z

**Published:** 2024-02-23

**Authors:** Subin Heo, Hyo Jung Park, Hyoung Jung Kim, Jung Hoon Kim, Seo Young Park, Kyung Won Kim, So Yeon Kim, Sang Hyun Choi, Jae Ho Byun, Song Cheol Kim, Hee Sang Hwang, Seung Mo Hong

**Affiliations:** 1 Department of Radiology and Research Institute of Radiology, Asan Medical Center, University of Ulsan College of Medicine, 88 Olympic-ro 43-gil, Songpa-gu, 05505 Seoul, Republic of Korea; 2 Department of Radiology, Seoul National University Hospital, 101 Daehangno, Jongno-gu, 110-744 Seoul, Republic of Korea; 3 Department of Statistics and Data Science, Korea National Open University, Seoul, Republic of Korea; 4 Division of Hepatobiliary and Pancreas Surgery, Asan Medical Center, University of Ulsan College of Medicine, Seoul, Republic of Korea; 5 Department of Pathology, Asan Medical Center, University of Ulsan College of Medicine, Seoul, Republic of Korea

**Keywords:** Pancreas, Neuroendocrine tumors, Radiomics, Multidetector computed tomography, Survival

## Abstract

**Background:**

Surgically resected grade 1–2 (G1-2) pancreatic neuroendocrine tumors (PanNETs) exhibit diverse clinical outcomes, highlighting the need for reliable prognostic biomarkers. Our study aimed to develop and validate CT-based radiomics model for predicting postsurgical outcome in patients with G1-2 PanNETs, and to compare its performance with the current clinical staging system.

**Methods:**

This multicenter retrospective study included patients who underwent dynamic CT and subsequent curative resection for G1–2 PanNETs. A radiomics-based model (R-score) for predicting recurrence-free survival (RFS) was developed from a development set (441 patients from one institution) using least absolute shrinkage and selection operator-Cox regression analysis. A clinical model (C-model) consisting of age and tumor stage according to the 8th American Joint Committee on Cancer staging system was built, and an integrative model combining the C-model and the R-score (CR-model) was developed using multivariable Cox regression analysis. Using an external test set (159 patients from another institution), the models’ performance for predicting RFS and overall survival (OS) was evaluated using Harrell’s C-index. The incremental value of adding the R-score to the C-model was evaluated using net reclassification improvement (NRI) and integrated discrimination improvement (IDI).

**Results:**

The median follow-up periods were 68.3 and 59.7 months in the development and test sets, respectively. In the development set, 58 patients (13.2%) experienced recurrence and 35 (7.9%) died. In the test set, tumors recurred in 14 patients (8.8%) and 12 (7.5%) died. In the test set, the R-score had a C-index of 0.716 for RFS and 0.674 for OS. Compared with the C-model, the CR-model showed higher C-index (RFS, 0.734 vs. 0.662, *p* = 0.012; OS, 0.781 vs. 0.675, *p* = 0.043). CR-model also showed improved classification (NRI, 0.330, *p* < 0.001) and discrimination (IDI, 0.071, *p* < 0.001) for prediction of 3-year RFS.

**Conclusions:**

Our CR-model outperformed the current clinical staging system in prediction of the prognosis for G1–2 PanNETs and added incremental value for predicting postoperative recurrence. The CR-model enables precise identification of high-risk patients, guiding personalized treatment planning to improve outcomes in surgically resected grade 1–2 PanNETs.

**Supplementary Information:**

The online version contains supplementary material available at 10.1186/s40644-024-00673-z.

## Background

Pancreatic neuroendocrine tumors (PanNETs) comprise a heterogeneous group of tumors with diverse biological behavior [1]. The 2017 World Health Organization (WHO) classification categorized PanNETs into three grades (G1, G2, and G3), based on the Ki-67 and mitotic count [2]. G3 PanNETs, previously classified as a subset of neuroendocrine carcinoma (NEC), are recognized as a distinct prognostic entity from G1-2 PanNETs, typically associated with poorer outcomes [3]. Conversely, G1–2 PanNETs exhibit varying disease trajectories, with some tumors demonstrating indolent growth even suitable for active surveillance without surgery [4], while others develop early recurrence after successful tumor removal, necessitating additional therapeutic intervention [5]. The wide range of reported lymph node metastasis (7.8–29.9%), distant metastasis (6.7–34.0%) [6–9], and 5-year overall survival (OS, 58.4–89.1%) [7, 10] underscores the prognostic heterogeneity of these tumors. Hence, accurate individualized risk stratification of surgically treated G1–2 PanNETs is important for optimal planning of adjuvant treatment and surveillance.

The 8th American Joint Committee on Cancer (AJCC) staging system [11] is currently widely used as a prognostic tool for PanNETs. However, its prognostic performance in G1-2 PanNETs is limited, showing modest predictive value for OS and discriminative prognostic performance between tumors with different stages [12–17]. Given the ongoing debate surrounding the prognostic efficacy of the current staging system, the identification of additional accurate prognostic biomarker may enhance individualized risk stratification.

CT is a commonly used imaging modality for diagnosing and evaluating the extent of PanNETs. The distinct imaging characteristics of these tumors can potentially reflect their biological aggressiveness [18–22], making CT a promising tool for prognostication, which can be maximized by applying radiomics analysis [23]. Previous radiomics studies on PanNETs have mainly focused on discriminating the histologic grade [24–27] or differentiating PanNETs from other pancreatic tumors [28, 29], while the prognostic performance of radiomics for post-surgical outcomes in G1–2 PanNETs remains unexplored. Therefore, the aim of this study was to develop and externally validate radiomics-based prognostic model, comparing its performance to the current clinical staging system in patients with G1–2 PanNETs.

## Methods

This study was approved by the institutional review boards of the two participating tertiary academic hospitals and the need to obtain informed consent was waived because of the retrospective nature of the study. Our study complies with the Transparent Reporting of a multivariable prediction model for Individual Prognosis Or Diagnosis guidelines [30].

### Study patients

Consecutive patients who underwent curative-intent resection for G1–2 PanNETs in one tertiary academic hospital (Asan Medical Center) between January 2004 and May 2020 were used to develop the prognostic models (development set). The inclusion criteria were: (a) surgically confirmed G1 or G2 PanNET, and (b) preoperative dynamic CT performed within 30 days prior to surgery. The exclusion criteria were: (a) history of local or systemic treatment for PanNET prior to surgery, (b) palliative-intent surgery, (c) history of other malignancy, and (d) CT examination without arterial phase (AP) or portal venous phase (PVP), and suboptimal CT quality. For external validation, patients from a separate tertiary academic hospital (Seoul National University Hospital) who underwent surgery between May 2004 and October 2021 were enrolled as a test set, following the same inclusion and exclusion criteria as used for the development set.

### Endpoint

The primary endpoint was recurrence-free survival (RFS), defined as the time from surgery to recurrence or death, whichever occurred earlier [31]. The secondary endpoint was OS, defined as the time from surgery to death [31]. Postoperative follow-up was conducted until December 2021, and included regular clinical assessment and imaging studies every 3–12 months and when clinically indicated. Patients with neither recurrence nor death were censored at their last follow-up date.

### Clinicopathological data

Patient demographics, clinical data, and pathological data, including tumor size, histologic grade, invasion of adjacent organs, invasion of major vessels, resection margin status, and lymph node status, were collected. Pathology slides were reviewed by board-certified pathologists at each institution. Tumors were classified as G1 NET (mitotic rate < 2 mitoses per 10 high-power fields [HPFs] and Ki-67 index < 3%) or G2 NET (2–20 mitoses per 10 HPFs or Ki-67 index of 3–20%) [32]. Staging was performed according to the 8th AJCC staging system [11] and was based on data including lymph node and distant metastasis status and tumor size.

### Image acquisition

Dynamic contrast-enhanced CT scans, including AP and PVP images, were acquired using scanners with 16 or more multidetector rows. The CT parameters are listed in Supplementary Table 1. Iodinated contrast medium was injected intravenously at a rate of 2–5 mL/s. The total volume of iodinated contrast medium was determined according to patient body weight (approximate rate, 2 mL/kg; maximum 150 mL). AP images were obtained using a bolus tracking technique with a 10–20 s delay after the attenuation of the aorta reached 100 Hounsfield units (HU). PVP imaging was performed 70–80 s after the intravenous injection of the contrast agent or 30 s after the acquisition of AP images.

### CT radiomics analysis

#### Tumor segmentation

Details of the whole radiomics algorithm process is illustrated in the Supplementary Figure S1. One abdominal radiologist (9 years of experience) performed manual segmentation of the entire tumor by drawing a volume of interest (VOI) along the tumor margin on both axial AP and PVP images using an in-house software package (AsanJ; Asan Medical Center, Seoul, Korea), in both the development and test sets (Supplementary Figure S2). In cases of multiple tumors, the tumor with the highest grade or the tumor with the largest size (if the grade of all tumors was the same) was chosen. To assess inter-observer agreement, an independent abdominal radiologist (6 years of experience) drew tumor VOIs in 30 randomly selected patients. The radiologists were blinded to the patients’ clinicopathologic data.

#### Radiomics feature extraction

Using in-house software (AsanFEx, Asan Medical Center, Seoul, Korea) written in Matlab (Matlab R2015a, Mathworks), radiomics features were extracted from the segmented VOIs of AP and PVP images according to the standardized process proposed by the Imaging Biomarker Standardization Initiative (IBSI) [33]. Prior to feature extraction, CT images were resampled into a uniform voxel size of 1 × 1 × 3 mm, and image normalization and intensity discretization were performed (Supplementary Table 2). All extracted feature values were scaled and centered to a mean value of 0 and standard deviation of 1. A detailed description is provided in the Supplementary Material 1.

#### Radiomics score (R-score) development

A multi-step feature selection process was undertaken to overcome the problems of overfitting and multicollinearity present when modeling using high-dimensional radiomics features. First, unreliable features with inter-reader concordance correlation coefficients (CCCs) < 0.8 were removed. Second, using the correlation analysis [34], highly correlated features with Pearson correlation coefficients > 0.9 were considered redundant and removed. Least absolute shrinkage and selection operator (LASSO)-Cox regression was then applied to the remaining features in order to build the R-score. First, the models were trained under three conditions: (1) all features extracted from both AP and PVP images; (2) features extracted from AP images only; and (3) features extracted from PVP images only. The regression model using the features extracted from only AP images showed the highest predictive performance of the three models. Therefore, the R-score was developed using the features extracted from AP images.

#### Development and validation of the predictive models

Two preoperative models were developed to predict the RFS of the patients in the development set. First, the clinical model (C-model) was built using patient age and the 8th AJCC-based tumor stage. Age was included because of its potential influence on the prognosis (e.g., because of co-morbidities and frailty). Second, the clinical-radiomics model (CR-model) was developed by combining the C-model and the R-score. The prediction of RFS and OS was externally validated on the test set for all models.

### Statistical analysis

Independent sample *t*-tests and chi-squared tests or Fisher’s exact tests were used to compare data, as appropriate. Interobserver agreements for the extracted feature values from 30 randomly selected patients were evaluated using the intraclass correlation coefficient (ICC). In the development set, LASSO-Cox regression analysis was used to build the R-score for predicting RFS, with the tuning parameter λ selected using 10-fold cross validation. The C-model and CR-model to predict RFS were developed using multivariable Cox regression analysis. The predictive capabilities of the models for RFS and OS were evaluated using Harrell’s concordance index (C-index) [35] and time-dependent receiver operating characteristics (ROC) curve analysis. The calibration capabilities of the models were assessed using a calibration plot that compared the predicted versus the observed Kaplan-Meier estimates of 3-year RFS and OS. The incremental differences between the C-model and CR-model in the prediction of 3-year RFS and OS were calculated using net reclassification improvement (NRI) and integrated discrimination improvement (IDI) [36, 37]. Optimal cut-off for CR-model was determined as the point with highest sum of sensitivity and specificity for 3-year RFS and OS, with both sensitivity and specificity exceeding 60%. All statistical analyses were performed using R version 4.2.0 (The R Foundation for Statistical Computing). The following R packages were used: “caret” for correlation analysis, “lmtest” for logistic regression, “glmnet” for the LASSO Cox regression analysis, the “survival” and “timeROC” packages to implement the Kaplan–Meier and ROC analysis, respectively, and the “survIDINRI” package to evaluate models’ incremental value. *P* values < 0.05 were considered statistically significant.

## Results

### Patient characteristics

**Fig. 1:**
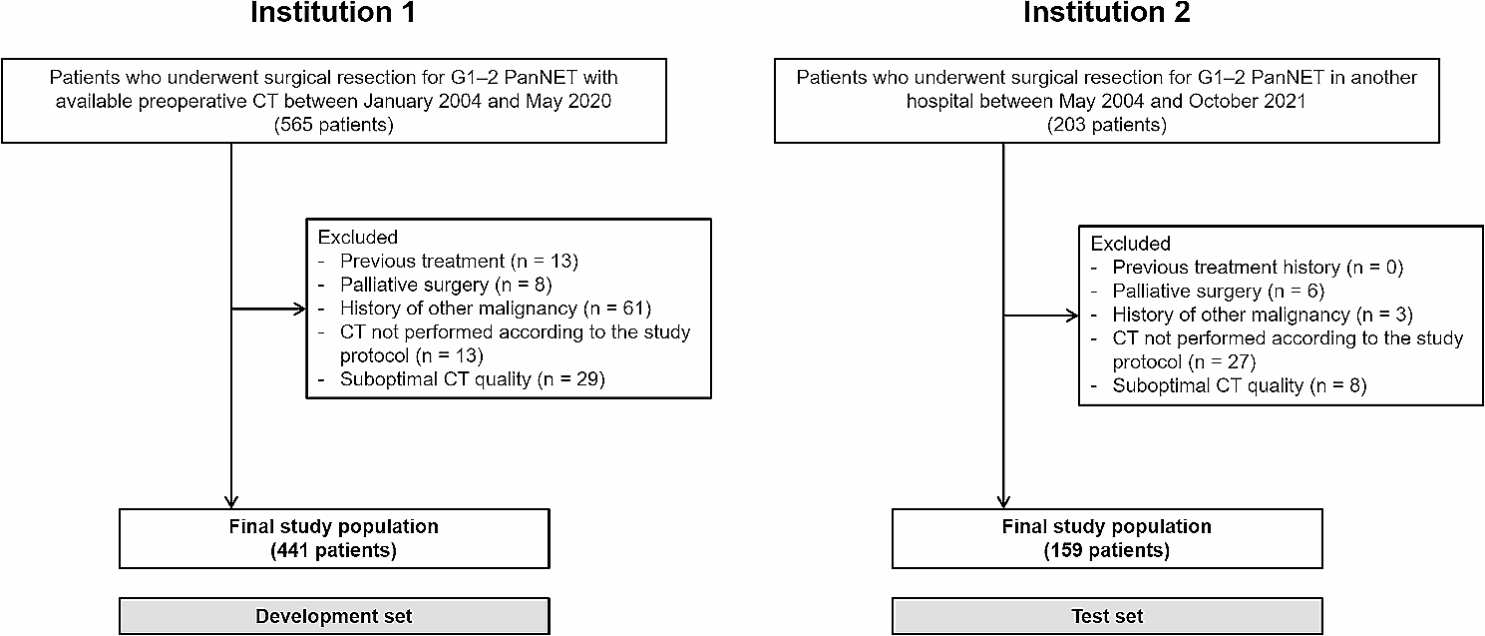
Flowchart of the study population. G1–2, grade 1–2; PanNET, pancreatic neuroendocrine tumor

The patient recruitment process is shown in Fig. 1. The development set included 441 patients (mean age 53.0 ± 12.3 years, 257 women) and the test set included 159 patients (mean age 55.9 ± 12.5 years, 83 women). The baseline patient characteristics are listed in Table 1. The distribution of AJCC stage did not differ significantly between the two sets (*p* = 0.327). The most common AJCC stage was stage II in the development set (48.8%; 215/441) and stage I in the test set (47.8%; 76/159). There were 12 (2.7%) stage IV patients in the development set and 3 (1.9%) in the test set. 19 patients (4.3%) in the development set and 4 patients (2.5%) in the test set had multiple tumors. The median follow-up periods were 68.3 months in the development set and 59.7 months in the test set. The median RFS and OS were not reached in either dataset. During follow-up, 58 patients (13.2%) experienced tumor recurrence and 35 patients (7.9%) died in the development set, and 14 patients (8.8%) experienced tumor recurrence and 12 patients (7.5%) died in the test set. Among the patients with tumor recurrence, rate of local recurrence, lymph node metastasis, and distant metastasis were 17.2%, 10.3%, and 72.4% in the development set, and 14.3%, 14.3%, and 71.4% in the test set, respectively. The rates of recurrence and death did not show any significant differences between the two sets (*p* = 0.463 for recurrence; *p* = 0.876 for death).

**Table 1:** Baseline clinical characteristics of the study patients

Variable	Development set	Test set	*P* value
Number of patients	441	159	
Age (years)*	53.0 ± 12.3	55.9 ± 12.5	0.011
Sex			0.186
Men	184 (41.7)	76 (47.8)	
Women	257 (58.3)	83 (52.2)	
Tumor size			0.061
< 2 cm	180 (40.8)	82 (51.6)	
2–4 cm	167 (37.9)	54 (34.0)	
> 4 cm	89 (20.2)	23 (14.5)	
2019 WHO grade			0.749
Grade 1	285 (64.6)	105 (66.0)	
Grade 2	156 (35.4)	54 (34.0)	
8th AJCC stage			0.327
I	175 (39.7)	76 (47.8)	
II	215 (48.8)	61 (38.4)	
III	39 (8.8)	19 (11.9)	
IV	12 (2.7)	3 (1.9)	
Follow-up data			
Follow-up (months)^†^	68.3 (38.9, 107.9)	59.7 (32.2, 101.0)	0.017
Recurrence	58 (13.2)	14 (8.8)	0.148
Death	35 (7.9)	12 (7.5)	0.876

Note—Unless stated otherwise, data are number of patients with percentages in parentheses

*Data are mean ± standard deviation

^†^Data are median with interquartile range in parentheses

AJCC, American Joint Committee on Cancer; WHO, World Health Organization

### R-score

A total of 692 radiomics features were extracted. After removing unreliable and redundant features (347 features with CCC < 0.8 and 274 features with pairwise correlation coefficient > 0.9), 71 features were included in the R-score development. Fifteen features extracted from the AP images were selected in the LASSO-Cox regression and were included in the final R-score: two morphologic features, four first-order features, four texture features, and five higher-order features (Table 2). Specifically, two morphologic features were volume (sum of all voxels included in the segmented VOI) and flatness (ratio of the major and least axis lengths). Four first-order features included intensity-based statistical features (maximum, 25th percentile, median, and robust mean absolute deviation, reflecting the overall distribution of the grayscale intensity of the tumor. Four texture features comprised cluster shade (reflecting skewness and uniformity), difference entropy and joint entropy (both reflecting the randomness/variability of grayscale intensity), and large zone high gray level emphasis (reflecting higher grayscale intensity and coarser textures), emphasizing the spatial heterogeneity of intra-tumoral grayscale intensity. Five higher-order features were extracted as textural features after applying either a Gaussian or Laplacian-of-Gaussian filter. These features were cluster shade, contrast (reflecting the dynamic range of gray levels), zone size variance, large distance high gray level emphasis, and high dependence high gray level emphasis, also reflecting spatial heterogeneity of intra-tumoral grayscale intensity of the tumor.

**Table 2:** Radiomics score (R-score)

Feature group	Radiomics feature*	Coefficient
Morphologic feature	Volume	–0.047
	Flatness	–0.075
First-order, intensity-based statistical feature	Maximum (AP, N)	–0.027
	25th percentile (AP, N)	–0.196
	Median (AP, LOG)	0.049
	Robust mean absolute deviation (AP, LOG)	–0.069
Texture, GLCM	Cluster shade (AP, N)	0.085
	Difference entropy (AP, N)	–0.106
	Joint entropy (AP, N)	0.656
Texture, GLSZM	Large zone high gray level emphasis (AP, N)	0.104
Higher order, GLCM	Cluster shade (AP, LOG)	0.251
	Contrast (AP, LOG)	0.028
Higher order, GLSZM	Zone size variance (AP, LOG)	–0.013
Higher order, GLDZM	Large distance high gray level emphasis (AP, G)	0.116
Higher order, NGLDM	High dependence high gray level emphasis (AP, LOG)	–0.112

Note—radiomics score is expressed as the sum of each radiomics feature multiplied by its coefficient

*The information within parentheses indicates the scan phases and types of images used for radiomics feature extraction

AP, arterial phase; G, Gaussian; GLCM, gray-level co-occurrence matrix; GLDZM, gray-level distance-zone matrix; GLSZM, gray-level size-zone matrix; LOG, Laplacian-of-Gaussian; N, non-transformed raw image; NGLDM, neighboring gray-level dependence matrix

The interobserver agreements for the R-scores obtained from the feature values extracted from the 30 VOIs independently drawn by the two radiologists were excellent (ICC, 0.96; 95% CI, 0.890–0.983). The C-index of the R-score for predicting RFS was 0.778 (95% CI, 0.748–0.808) in the development set and 0.716 (95% CI, 0.659–0.772) in the test set. For predicting OS, the R-score showed a C-index of 0.648 (95% CI, 0.588–0.709) in the development set and 0.674 (95% CI, 0.597–0.751) in the test set.

### Predictive models: C-model and CR-model

The results of the multivariable Cox regression analyses applied to the development set for building the two predictive models (C-model and CR-model) are shown in Table 3. In the construction of the CR-model, the R-score was revealed as a significant predictive factor for RFS (hazard ratio [HR], 3.681 [95% CI, 2.353–5.759; *p* < 0.001]). AJCC stage was also a significant predictor for RFS in both the C-model and the CR-model.

**Table 3:** Multivariable Cox proportional hazards models for predicting post-operative outcomes in the development set

Outcome	Variable	C-model		CR-model	
		HR (95% CI)	*P* value	HR (95% CI)	*P* value
Recurrence-free survival					
	Age	1.005 (0.985–1.025)	0.626	1.004 (0.984–1.024)	0.703
	AJCC stage		< 0.001		< 0.001
	I	1 (Ref)		1 (Ref)	
	II	2.989 (1.422–6.286)	0.004	0.881 (0.369–2.107)	0.777
	III	9.542 (4.166–21.854)	< 0.001	2.947 (1.176–7.386)	0.021
	IV	17.288 (6.602–45.269)	< 0.001	4.862 (1.725–13.709)	0.003
	R-score	–	–	3.681 (2.353–5.759)	< 0.001
Overall survival					
	Age	1.054 (1.021–1.088)	0.001	1.057 (1.023–1.092)	0.001
	AJCC stage		0.011		0.101
	I	1 (Ref)		1 (Ref)	
	II	1.408 (0.631–3.144)	0.404	0.609 (0.222–1.668)	0.334
	III	3.224 (1.164–8.930)	0.024	1.264 (0.387–4.124)	0.698
	IV	7.509 (2.344–24.058)	0.001	2.564 (0.670–9.814)	0.169
	R-score	–	–	2.296 (1.322–3.989)	0.003

AJCC, American Joint Committee on Cancer; C-model, clinical model; CR-model, clinical-radiomics model; CI, confidence interval; HR, hazard ratio; R-score, radiomics score

### Comparison of the predictive models’ performance

**Fig. 2:**
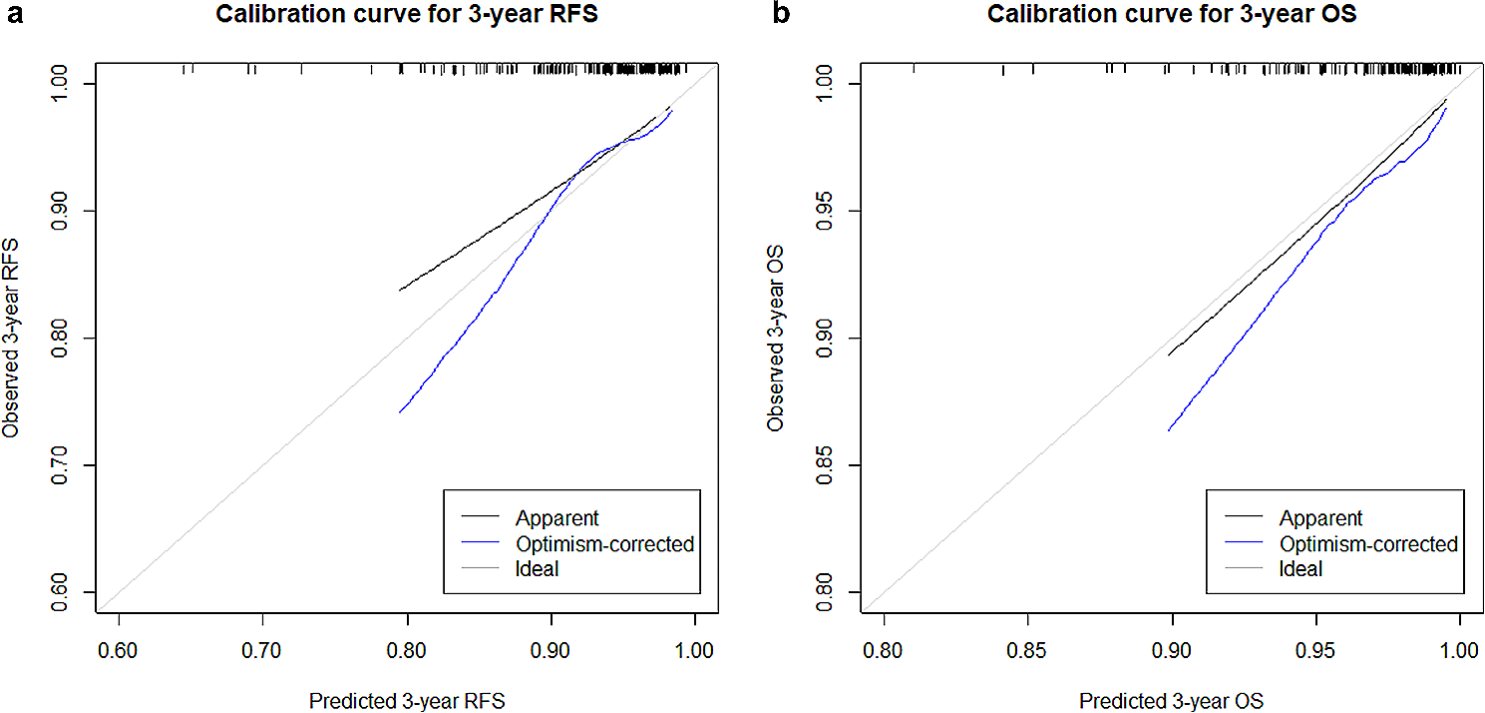
Calibration plots of the CR model showing the predicted and observed probability of (**a**) 3-year RFS and (**b**) 3-year OS in the test set. CR, clinical-radiomics; OS, overall survival; RFS, recurrence-free survival

**Fig. 3:**
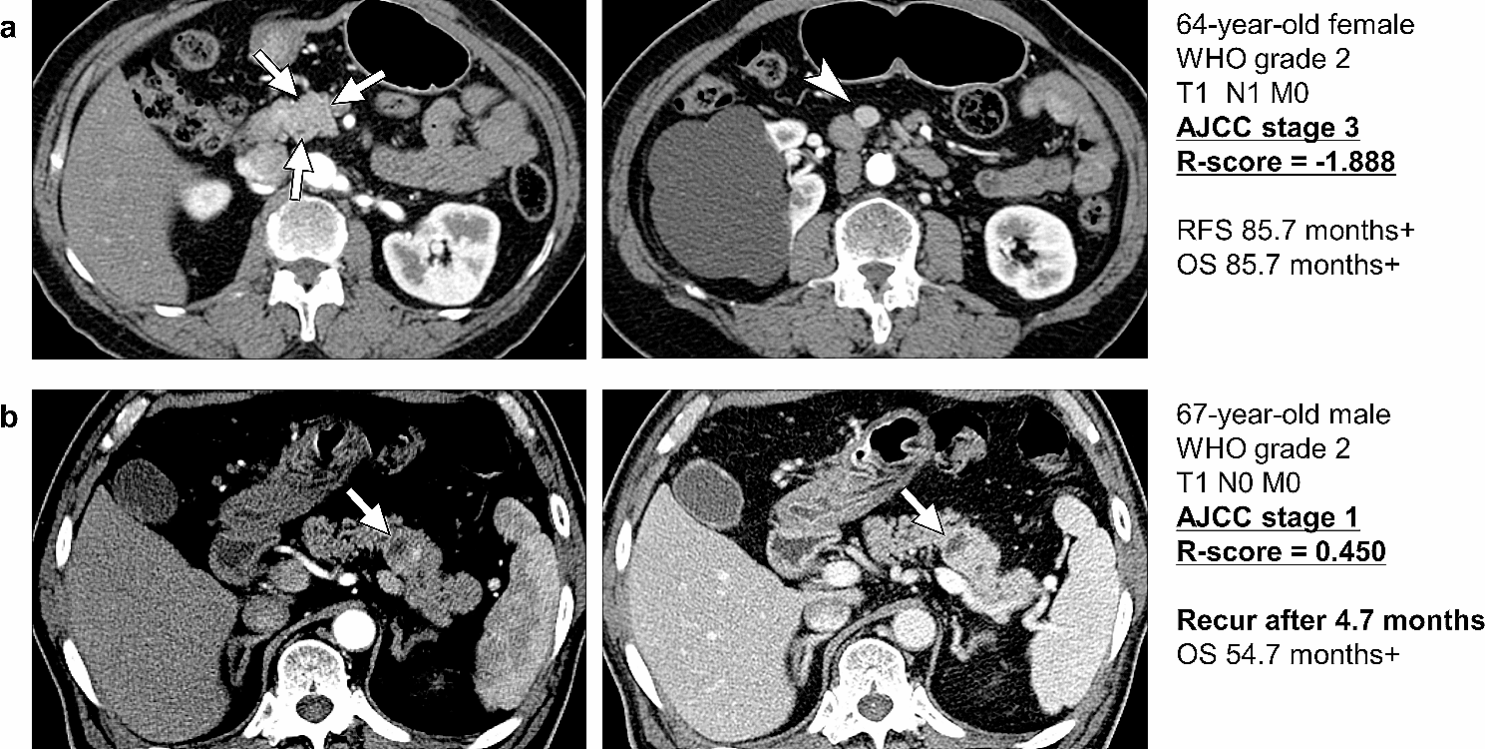
Representative cases of PanNET patients. (**a**) Preoperative AP CT images of a 64-year-old woman show a 1.8-cm homogeneously enhancing mass at the head of the pancreas (arrows). Regional lymph node metastasis is noted (arrowhead). This tumor was a WHO grade 2 PanNET with AJCC stage III. Although the initial tumor stage was high, the patient survived without recurrence until the last follow-up date (85.7 months after surgery). The R-score of this tumor was − 1.888. (**b**) Preoperative AP and PVP CT images of a 67-year-old man show a 1.9-cm mass at the tail of the pancreas. The mass shows heterogeneous enhancement, especially on AP images (arrows). This tumor was a WHO grade 2 PanNET with AJCC stage I. Despite the low AJCC stage, recurrence occurred at 4.7 months after surgery. The R-score of this tumor was 0.450, obviously higher than that of the patient in (**a**). AJCC, American Joint Committee on Cancer staging system; AP, arterial phase; OS, overall survival; PanNET, pancreatic neuroendocrine tumor; PVP, portal venous phase; RFS, recurrence-free survival; R-score, radiomics score; WHO, World Health Organization

The performance of each model in the prediction of postoperative outcomes is shown in Table 4. On the test set, the C-index of the CR-model for predicting RFS was 0.734 (95% CI, 0.674–0.793), which was significantly higher than that of the C-model (0.662 [95% CI, 0.591–0.733]; *p* = 0.012). The C-index of the CR-model in the prediction of OS was 0.781 (95% CI, 0.721–0.841), which was also significantly higher than that of the C-model (0.675 [95% CI, 0.605–0.745]; *p* = 0.043). In the prediction of RFS, the CR-model created by adding the R-score to the C-model achieved significant NRI (3-year NRI, 0.330 [95% CI, 0.163–0.479], *p* < 0.001) and IDI (3-year IDI, 0.071 [95% CI, 0.027–0.126], *p* < 0.001) over the C-model. However, the 3-year NRI (0.083, 95% CI, − 0.142–0.415; *p* = 0.365) and IDI (0.001 (95% CI, − 0.014–0.018; *p* = 0.824) for predicting OS were not significant. Calibration curves of the CR-model for predicting 3-year RFS and OS demonstrated good correlation between predicted and observed probability (Fig. 2). Time-dependent areas under the ROC curves (AUC) for each of the prediction models are shown in Supplementary Table 3. With a cutoff of − 0.23, the CR-model showed sensitivity and specificity of 81.4% and 72.3%, respectively, for predicting 3-year RFS, and 80.1% and 62.1%, respectively, for predicting 3-year OS. Representative cases are presented in Fig. 3.

**Table 4:** Performance of the R-score, C-model, and CR-model in predicting post-operative outcomes

Outcome	Model	Development set	*P* value	Test set	*P* value
		C-index (95% CI)	C vs. CR model	C-index (95% CI)	C vs. CR model
Recurrence-free survival					
	R-score	0.778 (0.748–0.808)		0.716 (0.659–0.772)	
	C-model	0.761 (0.732–0.790)		0.662 (0.591–0.733)	
	CR-model	0.811 (0.780–0.842)	0.045	0.734 (0.674–0.793)	0.012
Overall survival					
	R-score	0.648 (0.588–0.709)		0.674 (0.597–0.751)	
	C-model	0.697 (0.638–0.755)		0.675 (0.605–0.745)	
	CR-model	0.730 (0.673–0.787)	0.127	0.781 (0.721–0.841)	0.043

C-index, concordance index; CI, confidence interval; C-model, clinical model; CR-model, clinical-radiomics model; R-score, radiomics score

To compare the predictive performance of the radiomics-based model with the conventional imaging feature-based model, we developed a model based on the conventional imaging features including tumor margin, tumor heterogeneity, enhancement pattern, and tumor-to-parenchymal enhancement ratio on the arterial phase (Supplementary Material). In the test set, the conventional imaging feature-based model demonstrated a lower C-index (0.642, 95% CI 0.552–0.732 for RFS and 0.480, 95% CI 0.382–0.577 for OS) compared to the R-score (0.716, 95% CI 0.659–0.772 for RFS and 0.674, 95% CI 0.597–0.751 for OS). Additionally, a model combining both conventional imaging features and clinical features (age and AJCC) also showed a lower C-index (0.643, 95% CI 0.551–0.735 for RFS and 0.748, 95% CI 0.678–0.818 for OS) compared to the CR-model (0.734, 95% CI 0.674–0.793 for RFS and 0.781, 95% CI 0.721–0.841 for OS) in the test set.

## Discussion

In this study, we demonstrated the prognostic performance of radiomics-based models in patients who underwent resection for G1–2 PanNETs. The prognostic performance of a R-score derived from the preoperative dynamic CT images was validated in an external cohort, for which it showed a high C-index and significant incremental value for predicting RFS when added to a C-model. Our study indicates the potential of dynamic CT radiomics analysis as a preoperatively-available prognostic tool for predicting postsurgical outcomes in patients with G1–2 PanNETs.

In our study, G1–2 PanNETs showed variable tumor stages and clinical behaviors, consistent with previous reports, implying the necessity for more sophisticated and accurate prognostic tools than the current staging and grading systems. Although PanNETs are known for their heterogeneous composition, even within a single tumor [38, 39], the WHO grade is based on the highest mitotic or Ki-67 index, known as the “hot spot”, which reflects only a part of the tumor. To the contrary, radiomics analysis incorporates data from all parts of the tumor without a sampling bias, and should provide more meaningful information for disease prognostication than the WHO grading system. Furthermore, the second-order and higher-order radiomics features contain information about the relationships between neighboring pixels, thereby providing information on the complexity and spatial heterogeneity of all areas of the tumor [40]. Therefore, radiomics analysis should enable better disease prognostication than tumor grade alone, as we demonstrated in this study.

Among the 15 selected radiomics features, ‘joint entropy’ (belonging to texture features) and ‘cluster shade’ (belonging to texture and higher order features) were the features showing the highest positive coefficients. Both features reflect the spatial heterogeneity of intra-tumoral grayscale intensity, with joint entropy representing randomness in neighborhood intensity values, and cluster shade representing asymmetry about the mean value [40]. Other features reflecting tumor heterogeneity, such as ‘large zone high gray level emphasis’ (texture features) and ‘contrast’ and ‘large distant high gray level emphasis’ (higher order features) [40] also showed positive coefficients. By contrast, the morphologic and first-order features showed smaller coefficients than the texture and higher-order features, possibly indicating their lesser importance.

The C-model incorporating AJCC stage and age yielded a C-index of 0.662 for predicting RFS and 0.675 for predicting OS in the test set, values slightly higher than previously reported (0.65 for predicting OS when using the 8th AJCC stage alone) [13]. By combining the C-model and the R-score, the CR-model attained improved prognostic performance, yielding a C-index of 0.734 for predicting RFS and 0.781 for predicting OS. In addition, we demonstrated the incremental value of adding the R-score to the C-model. Our results suggest that AJCC stage and R-score can be used in combination to achieve improved pre-surgical prediction of survival outcomes in patients with G1–2 PanNETs and guide better individualized treatment.

Prior radiomics studies on PanNETs focused mainly on discriminating tumor grades; radiomics models in prior studies reached AUCs of 0.860–0.876 for discriminating G1 from G2 PanNETs [25, 27], and AUCs of 0.729–0.902 for discriminating G1 from G2–3 PanNETs [24, 26]. A few studies reported the prognostic value of CT features including size, lymph node metastases, hepatic metastases, enhancement pattern, and tumor-to-parenchymal enhancement ratio [22, 41–43]. Among these, the tumor-to-parenchymal enhancement ratio measured on PVP was reported as an independent predictor of RFS and OS in PanNENs (including both neuroendocrine tumors and carcinomas), and showed better prognostic performance than when measured on AP imaging [22]. However, for our R-score, only features extracted from AP imaging were selected, because model including the PVP features showed poor performance with beta-coefficient values of near zero. This difference may have stemmed from different study population, since G1-2 PanNETs are known to be more hypervascular compared to G3 PanNET or NEC [19, 42].

There are several limitations to our study. First, the retrospective study design may have introduced biases. We validated our results on an independent external test set to demonstrate the reproducibility and generalizability of our models. However, a prospective study may be needed to further validate the study results. Second, the relatively small number of patients with G2 PanNETs compared with those with G1 PanNETs resulted in a skewed tumor grade distribution in both the development and test sets. However, this study on the prognostic performance of CT-based radiomics in patients with G1–2 PanNETs is the largest of its kind to date, and may reflect the unbiased grade distribution encountered in clinical practice. Third, because of the long patient recruitment period, various CT scanners were used, which may have affected the image analysis. However, we believe that the variety of CT scanners and techniques used should increase the generalizability of our results and their applicability to other institutions. Fourth, the manual tumor segmentation was time-consuming and may limit reproducibility and clinical utility. With the recent advent of many deep learning-based tools for automatic segmentation of pancreatic tumors, there is potential for their application in our radiomics model. Future studies should actively incorporate these deep learning models to enhance the optimization and application of the radiomics model.

## Conclusions

In conclusion, we suggest the radiomics-based prediction of survival outcomes in patients with resected G1-2 PanNETs. The R-score was externally validated as a reliable predictor of both RFS and OS, and provided incremental value on predicting RFS when combined with a C-model. It may serve as a non-invasive prognostic tool for guiding individualized and optimized patient management in patients with G1-2 PanNETs.

### Electronic supplementary material

Below is the link to the electronic supplementary material.

Supplementary Material

## Data Availability

The datasets used and/or analyzed during the current study are available from the corresponding author on reasonable request.

## Abbreviations

PanNET: Pancreatic neuroendocrine tumors
RFS: Recurrence-free survival
OS: Overall survival
NRI: Net reclassification improvement
IDI: Integrated discrimination improvement
WHO: World Health Organization
NEC: Neuroendocrine carcinoma
AJCC: American Joint Committee on Cancer
AP: Arterial phase
PVP: Portal venous phase
HPF: High-power fields
HU: Houndsfield unit
VOI: Volume of interest
CCC: Concordance correlation coefficients
LASSO: Least absolute shrinkage and selection operator
ROC: Receiver operating characteristics
