# Supplementary material for: Prognostic value of CT-based radiomics in grade 1–2 pancreatic neuroendocrine tumors
Source: Cancer Imaging. 2024 Feb 23;24:28. doi: 10.1186/s40644-024-00673-z (PMC10885493; doi:10.1186/s40644-024-00673-z)
Supplement: Supplementary file 1 — Supplementary Material [file 40644_2024_673_MOESM1_ESM.docx]

**Supplementary Material**

**Extracted radiomics features**

The extracted feature classes included morphological features, first-order features, texture features, and higher-order features extracted using Gaussian and Laplacian-of-Gaussian filters.

*Morphological features*

The morphological features were shape-based features of the VOI. Thirteen morphological features were extracted from each of the non-filtered AP and PVP images.

*First-order features*

First-order features are features reflecting the distribution pattern of gray-level values within the VOI as a whole, but not addressing the spatial relationships between the pixels. The first-order features included two local intensity features and 20 intensity-based statistical features. A total of 22 first-order features were extracted from each of the non-filtered AP and PVP images.

*Second-order features (texture features)*

Second-order features are those reflecting the spatial relationship between an individual pixel and its neighboring pixels. Second-order features included 28 gray-level co-occurrence matrix (GLCM) features, 12 gray-level run length matrix (GLRLM) features, 16 gray-level size-zone matrix (GLSZM) features, 16 gray-level distance-zone matrix (GLDZM) features, and 17 neighboring gray-level dependence matrix (NGLDM) features; thus, a total of 89 features were extracted for each AP and PVP image.

All texture matrices were rotationally and translationally invariant. We extracted texture features using a two-dimensional approach with voxel intensity discretization using a fixed bin number of 32. Texture matrices for a VOI were computed by image section, and the GLCM and GLRLM were computed in four directions (0°, 45°, 90°, and 135°) for each image section. All directional and sectional matrices were merged together to generate a single matrix (i.e., one GLCM, GLRLM, GLDZM, and NGLDM) for a VOI. The texture features were extracted from the final merged matrix.

GLCM features reflect the frequency of two neighboring pixels with specific gray-level values, whereas GLRLM features assess run lengths, defined as the length of a consecutive sequence of pixels with the same grey level. GLSZM counts the number of groups of linked pixels, where the pixels are linked if the neighboring pixel has an identical discretized gray level. GLDZM counts the number of groups of linked voxels that share a specific discretized gray level value and possess the same distance to the edge of the region of interest (ROI), thereby capturing the relationship between location and gray level. NGLDM is defined as an alternative to GLCM, and aims to capture the coarseness of the overall texture and is rotationally invariant.

*Higher-order features*

Higher-order features are features extracted from filtered images. Gaussian and Laplacian-of-Gaussian filters were used to extract 22 first-order features and 89 second-order features from both AP and PVP images, resulting in a total of 444 higher-order features. Gaussian filtering has a smoothing effect that reduces image noise. Laplacian-of-Gaussian filtering involves a combination of Gaussian and Laplacian filters (an edge-enhancing filter), thereby allowing edge enhancement without unnecessary amplification of image noise.

**Comparison with model based on conventional imaging features**

CT scans of all patients in both the development and test sets were reviewed by a board-certified abdominal radiologist who was blinded to the patient clinical information and outcomes. Following findings were assessed: tumor margin, tumor heterogeneity, enhancement pattern and tumor-to-parenchymal enhancement ratio on the arterial phase. Enhancement pattern was categorized as either early enhancement and washout, persistent enhancement, or progressive enhancement. Early enhancement and washout was defined as tumor showing peak enhancement on arterial phase, with attenuation of the tumor decreasing more than 20 HU on portal venous phase. Persistent enhancement was defined as attenuation on portal venous phase within 20 HU range from the attenuation on arterial phase, and tumor was considered to show progressive enhancement when attenuation on portal venous phase was higher than 20HU compared to arterial phase. In the development set, the conventional imaging feature-based model demonstrated a lower C-index (0.712, 95% CI 0.681-0.744 for RFS and 0.642, 95% CI 0.586-0.697 for OS) compared to the R-score (0.778, 95% CI 0.748-0.808 for RFS and 0.648, 95% CI 0.588-0.709 for OS). A model combining both conventional imaging features and clinical features (age and AJCC) also showed a lower C-index (0.785, 95% CI 0.756-0.814 for RFS and 0.703, 95% CI 0.640-0.766 for OS) compared to the CR-model (0.811, 95% CI 0.780-0.842 for RFS and 0.730, 95% CI 0.673-0.787 for OS) in the development set.

**Supplementary Table 1. CT imaging techniques**

| **CT technique** | **Development set**  **(n = 441)** | | **Test set**  **(n = 159)** | | ***P* value** |
| --- | --- | --- | --- | --- | --- |
| CT detector configuration |  |  |  |  | 0.182 |
| 16–64 detectors | 171 | (38.8) | 52 | (32.7) |  |
| ≥ 64 detectors | 270 | (61.2) | 107 | (67.3) |  |
| Tube voltage |  |  |  |  | 0.005 |
| 80–110 kVp | 71 | (16.1) | 27 | (17.0) |  |
| 120 kVp | 370 | (83.9) | 128 | (80.5) |  |
| 130–140 kVp | 0 | (0) | 4 | (2.5) |  |
| Slice thickness |  |  |  |  | <0.001 |
| ≤ 2.5 mm | 122 | (27.7) | 49 | (30.8) |  |
| 3.0 mm | 297 | (67.3) | 87 | (54.7) |  |
| 3.2–5.0 mm | 21 | (4.8) | 23 | (14.5) |  |
| 7.0 mm | 1 | (0.2) | 0 | (0) |  |
| Pixel size |  |  |  |  | 0.020 |
| 0.4–0.7 mm | 324 | (73.5) | 101 | (63.5) |  |
| ≥ 0.7 mm | 117 | (26.5) | 58 | (36.5) |  |
| CT vendors |  |  |  |  | <0.001 |
| Siemens | 306 | (69.4) | 61 | (38.4) |  |
| GE | 128 | (29.0) | 15 | (9.4) |  |
| Philips | 6 | (1.4) | 71 | (44.7) |  |
| Others* | 1 | (0.2) | 12 | (7.5) |  |

Data are numbers with percentages in parentheses.

*Including Toshiba, and Philips and Neusoft Medical Systems (PNMS).

**Supplementary Table 2. Parameter configurations for image processing**

| **Parameter** | **Configuration** |
| --- | --- |
| Slice-wise (2D) or single volume (3D) | 2D |
| Interpolation | Yes |
| Resampled voxel spacing (mm) | 1 × 1 × 3 |
| Interpolation method | Tricubic |
| Intensity rounding | Nearest integer |
| ROI interpolation method | Tricubic |
| ROI partial mask volume | 0.5 |
| Normalization |  |
| Range (HU) | Mean ± 3 × standard deviation |
| Outlier filtering | None |
| Discretization |  |
| Texture and intensity histogram | Fixed bin number, 32 bins |
| First-order features | None |
| Texture |  |
| GLCM, NGLDM distance | 1 |
| GLSZM, GLDZM linkage distance | 1 |
| NGLDM coarseness | 0.0 |

ROI, region of interest; HU, Hounsfield unit; GLCM, gray-level co-occurrence matrix; GLSZM, gray-level size-zone matrix; GLDZM, gray-level distance-zone matrix; NGLDM, neighboring gray-level dependence matrix

**Supplementary Table 3. Time-dependent area under the receiver operating characteristics curves (AUC) on the test set for each prediction model**

| **AUC of the models** | **C-model** | **CR-model** |
| --- | --- | --- |
| Recurrence-free survival |  |  |
| 1-year | 0.727 (0.494–0.960) | 0.815 (0.605–1.026) |
| 3-year | 0.750 (0.605–0.896) | 0.780 (0.638–0.922) |
| 5-year | 0.612 (0.426–0.797) | 0.708 (0.566–0.850) |
| Overall survival |  |  |
| 1-year | 0.882 (0.829–0.935) | 0.951 (0.916–0.987) |
| 3-year | 0.629 (0.487–0.771) | 0.735 (0.581–0.890) |
| 5-year | 0.609 (0.385–0.833) | 0.743 (0.589–0.897) |

C-model, clinical model; CR-model, clinical-radiomics model


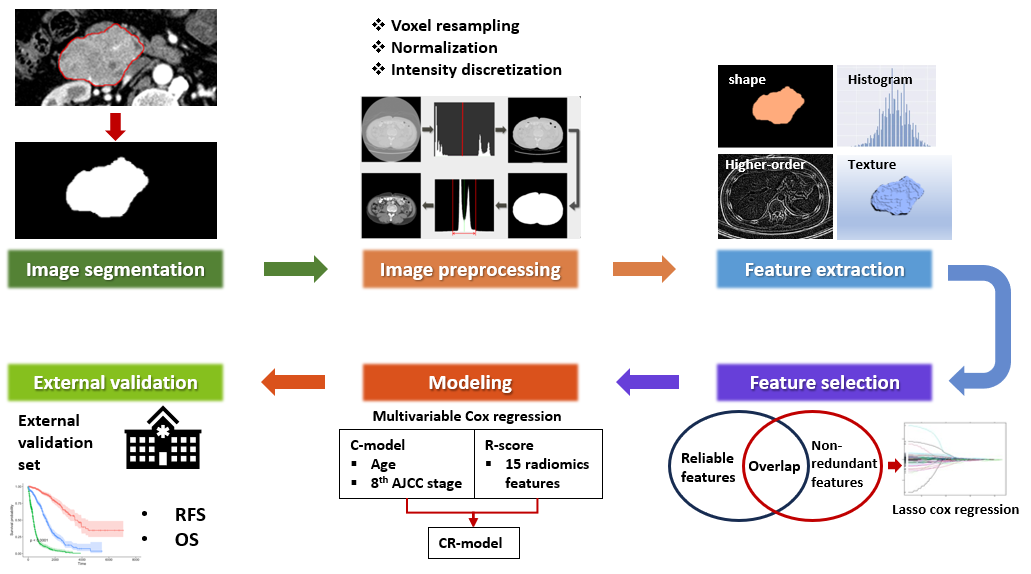


**Supplementary Fig. S1— Radiomics algorithm process.**

Tumor is manually segmented on the dynamic contrast enhanced CT scans. Segmented images are resampled into a uniform voxel size of 1x1x3 mm, and image normalization and intensity discretization are performed. Radiomics features are extracted, including shape, histogram, textural, and high-order features. Feature selection is performed after unreliable and redundant features are removed. Using the Least absolute shrinkage and selection operator (LASSO)-Cox regression, an R-score is developed. The clinical-model (C-model), including age and 8^th^ AJCC stage, is combined with the R-score to develop the clinical-radiomics model (CR-model). The performance of the models is validated in an external validation set.


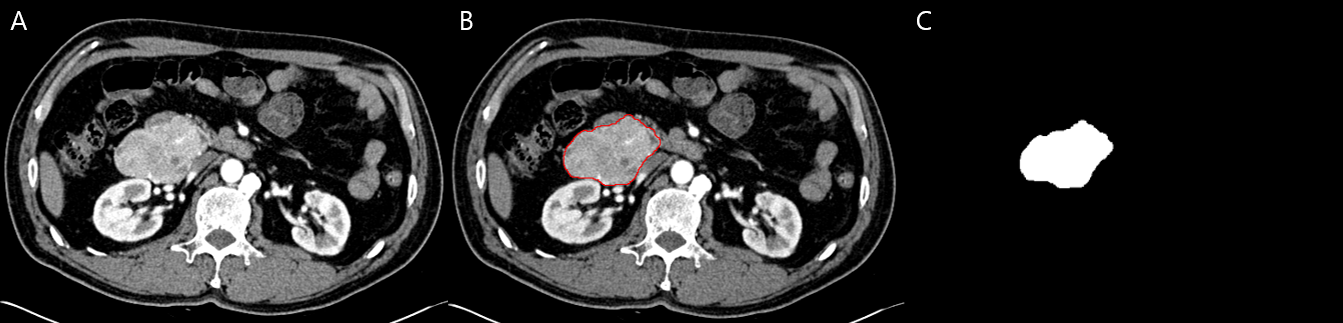


**Supplementary Fig. S2—** An example of tumor segmentation. Arterial phase CT scan of a 64-year old man shows an 8 cm heterogeneously enhancing tumor in pancreas head (A), which was confirmed as a WHO grade 2 pancreatic neuroendocrine tumor. Manual segmentation was performed along the tumor margin (B), and a segmentation mask was acquired (C).
